# A Mission Simulating the Search for Life on Mars with Automated Drilling, Sample Handling, and Life Detection Instruments Performed in the Hyperarid Core of the Atacama Desert, Chile

**DOI:** 10.1089/ast.2022.0055

**Published:** 2023-12-20

**Authors:** Carol R. Stoker, Brian J. Glass, Thomas R. Stucky, Arwen I. Dave, Linda T. Kobayashi, Richard C. Quinn, Mercedes Moreno-Paz, Laura Sánchez-García, Maria F. Mora, Florian Kehl, Víctor Parro, Peter A. Willis, Alfonso Davila, Eldar Noe Dobrea, Jon C. Rask, Daniel Ricardo

**Affiliations:** ^1^ NASA Ames Research Center, Moffett Field, California, USA.; ^2^ Centro de Astrobiología (CAB), CSIC-INTA, Madrid, Spain.; ^3^ Jet Propulsion Laboratory, California Institute of Technology, Pasadena, California, USA.; ^4^ Innovation Cluster Space and Aviation (UZH Space Hub), Air Force Center, University of Zurich, Zurich, Switzerland.; ^5^ Center for Theoretical Astrophysics and Cosmology, Institute for Computational Science, University of Zurich, Zurich, Switzerland.; ^6^ Institute of Medical Engineering, Space Biology Group, Lucerne University of Applied Sciences and Arts, Hergiswil, Switzerland.; ^7^ Space Technology and Industry Institute, School of Engineering, Swinburne University of Technoogy, Hawthorn, Victoria, Australia.

**Keywords:** Mars, Analog field experiment, Drilling, Life detection instruments, Atacama Desert

## Abstract

We report on a field demonstration of a rover-based drilling mission to search for biomolecular evidence of life in the arid core of the Atacama Desert, Chile. The KREX2 rover carried the Honeybee Robotics 1 m depth The Regolith and Ice Drill for Exploration of New Terrains (TRIDENT) drill and a robotic arm with scoop that delivered subsurface fines to three flight prototype instruments: (1) The Signs of Life Detector (SOLID), a protein and biomolecule analyzer based on fluorescence sandwich microarray immunoassay; (2) the Planetary *In Situ* Capillary Electrophoresis System (PISCES), an amino acid analyzer based on subcritical water extraction coupled to microchip electrophoresis analysis; and (3) a Wet Chemistry Laboratory cell to measure soluble ions using ion selective electrodes and chronopotentiometry. A California-based science team selected and directed drilling and sampling of three sites separated by hundreds of meters that included a light-toned basin area showing evidence of aqueous activity surrounded by a rocky desert pavement. Biosignatures were detected in basin samples collected at depths ranging from 20 to 80 cm but were not detected in the surrounding area. Subsurface stratigraphy of the units drilled was interpreted from drill sensor data as fine-scale layers of sand/clay sediments interspersed with layers of harder material in the basins and a uniform subsurface composed of course-to-fine sand in the surroundings. The mission timeline and number of commands sent to accomplish each activity were tracked. The deepest sample collected (80 cm) required 55 commands, including drilling and delivery to three instruments. Elapsed time required for drilling and sample handling was less than 3 hours to collect sample from 72 cm depth, including time devoted to recovery from a jammed drill. The experiment demonstrated drilling, sample transfer technologies, and instruments that accomplished successful detection of biomolecular evidence of life in one of the most biologically sparse environments on Earth.

## 1. Introduction

Searching for life on Mars will require access to the subsurface to sample materials that have been protected from organic destruction by ultraviolet (UV) light (Stoker and Bullock, 1997; Moores and Schuerger, 2012), cosmic rays (Kminek *et al*., 2003; Dartnell *et al.*, 2007; Pavlov *et al*., 2012), and other oxidation processes (Georgiou *et al.*, 2017). Upcoming and proposed missions hope to access the martian subsurface to search for evidence of life. The European ExoMars rover mission, currently planned to launch in 2028, will land in Oxia Planum and drill up to 2 meters to collect samples to be analyzed by a variety of instruments (Vago *et al*., 2017; Altieri *et al*., 2023) that search for signatures of a habitable environment and signs of life.

Other drilling missions are being proposed to search for life in martian ground ice. The Icebreaker mission (McKay *et al*., 2013) proposed to drill into high-latitude ground ice to search for biomolecular evidence of modern life. More recently, the 2022 Decadal Survey of Planetary Science (National Academies of Sciences, Engineering, and Medicine, 2022) called for drilling into midlatitude ground ice to search for evidence of life. These missions require robotic drilling capability operated from Earth with substantial time delays. The NASA Curiosity mission has provided flight experience with a shallow (5 cm) rock drill (Abbey *et al.*, 2019, 2020), but deep drilling represents a new space flight capability with risks that can impact mission success.

Field tests of drilling systems in high-fidelity analog environments are an important way to gain experience and prove out drilling systems that are destined for flight for relatively low cost. They also help to expose the scientific community to their capabilities and limitations.

In this article, we report on an end-to-end simulation of a robotic mission featuring a drill carried on a rover that sampled in a biologically sparse Mars analog environment to search for biosignatures of life. The field experiment was part of the Atacama Rover Astrobiology Drilling Studies (ARADS) project that took place in the Atacama Desert, Chile, the driest desert on Earth and a recognized Mars analog. ARADS was a NASA PSTAR project with science, engineering, and operational objectives. A broad overview of the ARADS project is presented by Glass *et al.* (2023). The goals of this field experiment were both scientific and operational with the desire to demonstrate the performance of the drilling, sample handling, and the life search protocols, including the analysis of samples with flight prototype onboard instruments. The experiment was performed as a mission simulation with sample selection, rover, and drill operations directed by a science team located in California during the test. This article provides an overview of the experiment with emphasis on the drilling, sample handling, and operational aspects, whereas other articles from the field experiment focus on the results from the life search instruments that were demonstrated in the test (Mora *et al*., 2020; Moreno-Paz *et al*., 2023).

Previous work with robotic drilling systems demonstrated in field tests in Mars analog environments includes the 2005 MARTE experiment (Stoker *et al*., 2008) where a drill capable of bringing cores from up to 10 m depth and analyzing them with onboard instruments was drilled to 6 m in Rio Tinto Spain. A remote science team inspected images of cores and selected subsamples for analysis by the Signs of Life Detector (SOLID) instrument (Parro *et al*., 2008). A later experiment, also performed in Rio Tinto, Spain, demonstrated robotic drilling using a 1 meter drill, sample collection with a robotic arm and scoop, and samples analyzed by a later prototype of the SOLID instrument (Sánchez-García *et al.*, 2020). While this latter experiment used many of the same drilling and sample handling hardware elements as the experiment reported here, the drilling was in moist sediments associated with sulfide-rich mine tailings. The payload was located on a mock-up of a stationary planetary lander modeled after the Phoenix and Insight landers, and all robotic operations were locally controlled.

A previous field experiment that is highly relevant to ours was performed as part of the LITA projects (Cabrol *et al*., 2007). Field work was conducted in 2013 in the hyperarid core of the Atacama Desert by a rover carrying a Honeybee Robotics drill (Zacny *et al*., 2014; Warren Rhodes *et al*., 2019) with direct lineage to The Regolith and Ice Drill for Exploration of New Terrains (TRIDENT) (Zacny *et al*., 2021). The rover traversed 50 km and drilled 32 holes to 80 cm depth. Samples were analyzed with an onboard Raman spectrometer instrument, and extensive ground truth analysis of collected drilled and nearby hand-dug pit samples was performed (Warren Rhodes *et al*., 2019).

Our field work was performed in September 2019 in the hyperarid core of the Atacama Desert in northern Chile, an area widely regarded as a Mars analog site (McKay *et al*., 2003). Conditions in this region have ranged between arid to semiarid since the late Jurassic (150 million years) but have been primarily hyperarid for the last 2 million years (Hartley and Chong, 2002; Hartley *et al.*, 2005; Jordan *et al*., 2014). Protracted periods of extreme aridity in the Atacama hyperarid core have created “Mars-like soils” (Navarro-González *et al*., 2003) that are ancient, extremely dry, chemically heterogeneous, and enriched in salts similar to those on Mars (Ewing *et al*., 2006; Amundson *et al.*, 2008).

These Atacama soils harbor some of the lowest levels of organic biomass and viable bacteria on Earth and have important implications for chemistry, habitability, and the search for organics and life on Mars (Navarro-González *et al*., 2003; Drees *et al*., 2008; Crits-Christoph *et al*., 2013). In one location, drilling into the subsurface (Wilhelm *et al.*, 2017) encountered layers of nearly pure halite underneath clay-rich layers overlain by sandy sediment reflecting a history of aqueous activity. Despite the extreme aridity, microorganisms have been reported in the soils that appear to be adapted to the desiccated conditions, the high salinity, and the high UV radiation environment (see Azua-Bustos *et al*., 2012 for review). *Polyextremophilic* microbial communities tolerant of high salts, extreme temperatures, and high solar irradiance also inhabit halite nodules in salt-rich playa deposits (Wierzchos *et al.*, 2006; Dávila *et al*., 2008, 2010).

While the long-term mean annual precipitation in the region is very low, environmental conditions are dynamic on timescales of hundreds to thousands of years, and century-scale rainfall events are an important agent of landscape evolution (Pfeiffer *et al*., 2021). Such events can produce or reactivate hillside streams that terminate in relatively small playas in topographic lows. After a period of weeks to several months following a rainfall event, the playa surfaces dry leaving behind light colored sediments. One of these small desiccated playas was selected to be the operational area of the ARADS field experiment.

## 2. Methods

### 2.1. Field site

**FIG. 1.**
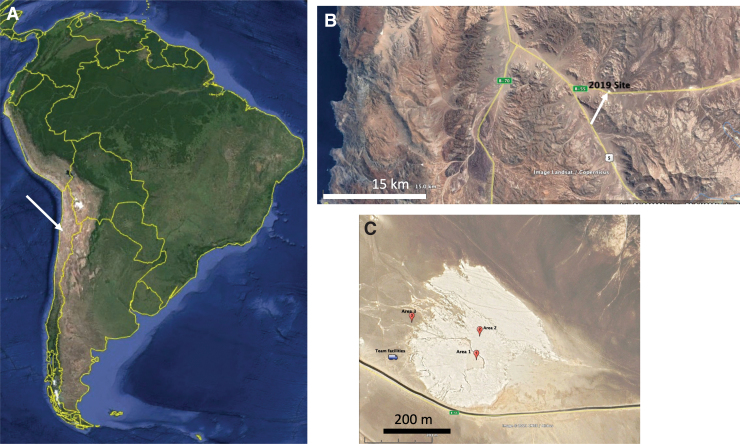
**(A)** Location of the Atacama core in northern Chile. The arrow points to the field area. **(B)** Road map with arrow pointing to field site. **(C)** Study area with location of three drill sites (red markers). Image credits: Google Earth. North is up in all figures.

The field site (Fig. 1) was chosen by the remote science team (RST) that supported the mission operation by examining satellite images of candidate sites displayed in Google Earth. The chosen site is located at −24.101642° latitude and −70.138175° longitude situated 67 km from the city of Antofagasta Chile and 22.4 km from the Yungay field camp in use by ARADS since 2014 (Glass *et al*., 2023). The ARADS team had not previously performed drilling or sample analysis in this area and chose it to perform an end-to-end blind-test of a mission-like rover carrying a 1 meter (1-m) drill and instrumentation to search for biomolecular evidence of life as both are relevant to future Mars missions.

### 2.2. Mission hardware and software

**FIG. 2.**
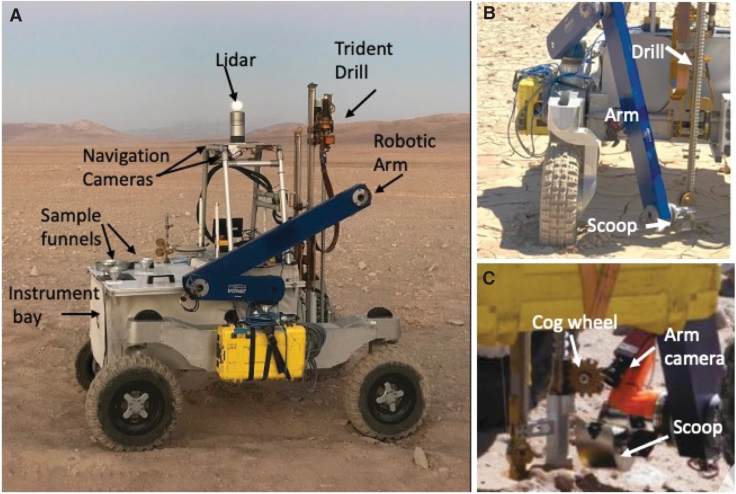
**(A)** KREX rover showing the main systems used in the test. The rover carried the TRIDENT drill and a robotic arm with scoop for sample capture. Three flight prototype instruments were housed within the rover's instrument bay. Funnels on the deck of the rover led to instrument inlets under the deck. **(B)** Robotic arm and scoop deployed for sample capture from the drill. Sample was acquired when the drill was withdrawn from the target depth. **(C)** Close-up of cog wheel that pushes sample off the auger flights and into the scoop.

All the robotic systems were integrated onto the KREX2 test bed rover (Fig. 2), a four-wheeled mobile robot designed to satisfy three goals: (1) autonomous movement at moderate speed (up to 1.5 m/s) across a variety of unstructured natural terrain; (2) low time to repair and high robustness; and (3) ability to support a wide range of field work tasks, including scouting, mapping, site preparation, and hosting instruments and sampling systems. KREX2 is comparable in physical footprint with the Mars Exploration Rovers (Spirit and Opportunity). The rover has independent, four-wheel drive, and all-wheel steering with a central rocker suspension that allows it to carry a 200 kg payload, comparable with the Mars Curiosity rover, over 30 cm obstacles and slopes up to 30°.

KREX2's standard sensors include a NovAtel differential GPS system, a Honeywell digital compass, two AVT Manta GigE cameras mounted with fixed pointing on a mast for imaging, a NovAtel inertial measurement unit, a Velodyne scanning lidar, and wheel encoders. KREX2 can navigate in an unprepared environment due to its sensor suite and avoid obstacles such as rocks, uneven terrain, or large slopes. It is controlled through VERVE software developed at NASA (Flückiger and Utz, 2014), with a full graphical user interface and navigation software that allows the topical mapping of the surroundings. These features enable the rover operator to give it high-level commands and let the rover operate autonomously.

An engineering model of a TRIDENT drill (Zacny *et al*., 2021) built by Honeybee Robotics (Fig. 2) was mounted on the KREX2 rover and provided subsurface samples to the instruments. TRIDENT is planned for flight to the Moon on the Volatiles Investigating Polar Exploration Rover (VIPER) mission (Colaprete *et al.*, 2020; Colaprete, 2021; Smith *et al*., 2022). TRIDENT was also selected for the proposed *Icebreaker* mission to search for modern life in Mars ice deposits (McKay *et al*., 2013).

The TRIDENT drill consists of several subsystems that include a rotary-percussive drill head for providing percussion and rotation of the drill string, a deployment stage to deploy the drill to the ground, a feed stage to advance the drill into the subsurface, a solid drill string with full-faced bit and auger flights for bringing drilled materials to the surface, and a cog wheel (Fig. 2C) interfaced to the auger flights that pushes off cuttings that stick to them. This latter system can be switched out for a circular brush. The drill produces a 24.5-mm-diameter hole up to 1-m deep with a single string. The percussive energy is 3.2 J/blow at a frequency of 16 blows per second. The auger rotation speed is 120 revolutions per minute. Its rotary and percussive actuators are 200 W each, and the maximum weight on bit (WOB; force pushing the drill down) is limited to 100 N to simulate drill deployment from a lightweight platform in low martian gravity. To control the WOB during the drilling process, a load cell is axially aligned with the drill segment to provide accurate feedback of drilling loads to the control system. The feedback from drilling sensors allows sensing of subsurface density changes at scales of ∼1 cm. When drilling sensors exhibit values that exceed preprogrammed thresholds, percussion is initiated. Drilling is accomplished using a bite-sampling approach where a small interval is drilled (nominal 10 cm), then the drill string is brought to the surface, and the material carried on the auger flights is scraped off where it can be captured by the scoop that can deliver samples to instruments for analysis.

If no sample is desired from that interval, the scoop can move the cuttings to a dump pile. This bite-sampling process simulates a martian drilling scenario (Zacny *et al.*, 2014) and is also planned for the VIPER mission to the moon.

Samples were transferred from the drill to the instruments via the sample-handling arm (Fig. 2B, C), a 4 degree-of-freedom 2-m length robotic arm from Maxar Incorporated. The arm has heritage from the NASA Phoenix and INSIGHT (Interior Exploration using Seismic Investigations, Geodesy and Heat Transport) Mars missions. A scoop with a motorized wiper blade at the rear is mounted on the wrist of the robotic arm (Fig. 2C). The scoop was designed to capture the material brushed off the auger by the cog wheel and to push sample aliquots into the three instruments without the need to reload the scoop between sample deliveries. When the scoop is placed below the cog wheel and the drill is retracted, cuttings fall into the scoop. The wiper blade pushes samples out of the scoop even if they are sticky.

One test objective was to determine how reliably multiple instruments could be fed with a single scoop of soil by progressively moving the wiper blade forward. A camera was also mounted on the arm near the wrist to image the sample in the scoop before delivery. The arm camera could also image the ground before drill deployment to see potential obstacles and image the drill string to determine whether cuttings were stuck to it. If the subsurface material is sufficiently sticky, similar to fine grained soils with high moisture content, the cog wheel does not completely remove it from the auger flights. Previous ARADS Atacama drilling tests found that subsurface soil in some areas was moist within 1-m of the surface and material was visible on the auger after the cog wheel had cleaned it.

### 2.3. Sample analysis instruments

Three analytical instruments for sample analysis were mounted in the rover body with sample delivery provided through funnels mounted on the deck. These instruments analyzed samples for biosignature compounds and soluble ions and were as follows.

(1) SOLID is a microarray-based immunosensor, developed at the Centro de Astrobiología (CAB) in Spain. It processes samples by extracting biomolecules into a liquid solution by ultrasonication, and then detects them using the fluorescent sandwich immunoassay. The instrument in different configurations has been used in a variety of previous field missions (Parro *et al.*, 2008, 2011a; Sánchez-García *et al*., 2020). Details of the design of the SOLID instrument can be found in the work of Parro *et al.* (2011b). Moreno-Paz *et al.* (2023) present the configuration used in this experiment.
(2) Planetary *In Situ* Capillary Electrophoresis System (PISCES) was another sample analysis instrument demonstrated in the test. It is a liquid-based analytical platform developed at the Jet Propulsion Laboratory (JPL) for end-to-end automated analysis of amino acids (Willis *et al*., 2015). PISCES is composed of two subsystems: the subcritical water extractor (Kehl *et al*., 2019) and the chemical laptop, a fully automated microchip electrophoresis analyzer utilizing laser-induced fluorescence detection (Mora *et al*., 2020).
(3) A Wet Chemistry Laboratory (WCL) cell was the third sample analysis instrument in the rover payload. The four-cell WCL was a component instrument flown on the 2007 Phoenix Mars Scout Mission as part of the MECA payload package (Kounaves *et al*., 2009). The WCL sensors measure conductivity, oxidation–reduction potential (Quinn et al., 2011), soluble ions (NH_4_^+^, Ba^+^, Br^−^, Ca^2+^, Cl^−^, I^−^, Li^+^, Mg^2+^, NO_3_^−^/ClO_4_^−^, K^+^, Na^+^, H using ion selective electrodes (Kounaves *et al*., 2009), a gold electrode for cyclic voltammetry, and platinum and silver electrodes for chronopotentiometry (CP). One objective of the ARADS WCL field tests was to demonstrate a novel CP technique using silver working electrodes for the specific quantification of both halides (anodic) and nitrate (cathodic), without potential interference from the presence of perchlorate. On the Phoenix mission, perchlorate interfered with the electrode meant to detect nitrate (Hecht *et al*., 2009).

### 2.4. Mission and science planning

Remote operations planning required definition of roles and responsibilities for remote science and field teams before and during the simulated mission operation. Roles were articulated for a flight lead, RST lead, and RST members who participated in tactical mission planning. The RST members were selected based on knowledge of relevant biology and geology in the Atacama. Each had experience studying comparable sites in Chile but not the specific site of the mission simulation. For each instrument, the RST included instrument scientist specialists who analyzed the data produced after each sample analysis.

In preparation for the field experiment, requirements for minimum, baseline, and full mission success were developed to guide the planning. Full mission success required that samples be collected and analyzed from three different locations in the field area, with each instrument conducting sample analysis in at least two sites with at least one sample acquired from 50 cm or greater depth. Next, a strategic plan was developed to identify all the operations needed to achieve full mission success. The time required for each operation, including driving to a target location; collecting images and panoramas; drilling, collecting, and delivering samples to the instruments; and sample analysis by each instrument, was determined from prior experience and engineering tests with the system. This information was used to develop a timeline for the mission that allowed for completion of the mission in six field days.

A command dictionary was created to specify the suite of commands needed to operate all the systems and the protocol for communication between the remote operations and field teams. This document provided a template for building daily commands sent by the RST to the field team. The tight mission timeline made it critical that all operations could be performed using this template.

Before shipping equipment to the field, a test was performed in an outdoor test yard at the NASA Ames Research Center (hereafter ARC) to validate the preplanned routines to be used in the field mission. Tests with the rover carrying the camera system, drill, and arm were used to develop arm joint positions needed to deliver sample to instruments and point the robotic arm camera to image the drill footprint area before drill deployment to assess potential hazards and image the scoop to verify a sample was acquired.

Each day of operation required driving the rover to a location and then drilling to acquire and analyze samples retrieved from a specific depth requested by the RST. At the end of each day's operation, the rover was driven to a shelter where it was securely stored overnight so each sample acquisition required a new drive and new hole to be drilled. Given the time required to perform drilling and sample collection and to operate all three analytical instruments, at least 14 h was needed to accomplish all the needed tasks. This required the field team to operate in two shifts. To accommodate the time needed for the PISCES sample analysis (7.25 h), it ran during the early morning on the sample that was acquired the previous day. Each day's new drilling and sample collection operation started after the PISCES analysis was completed.

**FIG. 3.**
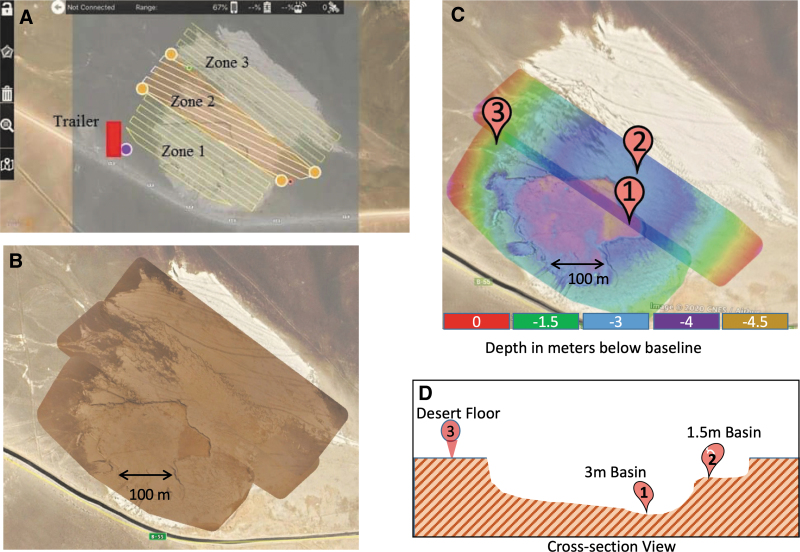
**(A)** Samples were collected within the area covered by the lines that show the route followed by drone flights over the field area. The rectangle represents field facilities housing people and equipment. **(B)** Satellite image overlaid by a merged drone-collected image product. **(C)** Digital elevation model produced from the drone flights; the numbers show the three locations drilled. Image products in **(B, C)** were made with commercial software MapsMadeEasy™. **(D)** Representation of the topography of the sampled areas (not to scale).

Before the field team deployment, the RST used satellite images of the field site (Fig. 1) to select three target areas for drilling that would characterize the area and test hypotheses developed about the site. These targets were incorporated into the initial strategic plan for the mission. While the light-toned “playa” area was easily identified in these images, the topography could not be determined. Immediately after the field team arrived in Chile, they flew a drone over the site in a grid pattern to collect images that were analyzed with commercial software (mapsmadeeasy.com) to produce a stitched high-resolution image of the area surveyed as well as a color-coded elevation model (Fig. 3).

The elevation model revealed a deeper basin (Area 1), nested within a shallower one (Area 2), with a scarp separating the two areas. Small-scale (approximately tens of centimeters) polygons covered the ground in Area 1, but were not present in Area 2. The darker toned region immediately outside the light-colored basin was designated Area 3. The RST then refined the strategic plan to update the drill targets based on the additional information provided by the drone flight. Figure 3C shows the final drill targets. Drill target 1 was selected to sample the deepest material exposed in Area 1. As this was the highest priority target, it was designated as the first target to drill and sample.

Drill target 2 was placed in Area 2, which was elevated above Area 1 and appeared more eroded and disturbed by human visitation (visible tire tracks). Finally, Area 3 was selected for drill target 3 to represent typical Atacama soil unmodified by aqueous activity.

The sampling strategy addressed the hypothesis that the basin areas have been periodically flooded by rainfall events that fill them to different levels, and microbial activity occurs in the ponded water and sediments. After the ponds evaporate, molecular biosignatures are left behind in the surface and subsurface sediments. The RST looked for evidence to evaluate the following hypothesis:

(1) If the drill string showed evidence of subsurface soil stuck to the auger flights after it returned to the surface, this would suggest that the subsurface was still moist from the recent rainfall events (rainfall events were noted in the Yungay region in 2015 and 2017, which resulted in ponded water in some basins, Azua-Bustos *et al*., 2018).
(2) A vertical profile of biomolecules obtained by drilling could indicate whether biosignature type or abundance varied with depth.
(3) Biomolecules should be elevated near the surface if microbial mats developed at the surface when water was present in the basin.
(4) Samples from Area 1 might show biosignatures in higher abundance than Area 2, since it was apparently flooded more recently. Area 3, undisturbed Atacama soil outside of the playa, was expected to have the lowest abundance of biosignatures of the three sites.

During the science mission operation, most RST members were located at NASA ARC in California. However, the instrument scientists for SOLID and PISCES were located at their home institutions (CAB in Spain and Jet Propulsion Laboratory in California, respectively) where they received and interpreted instrument data. The field team was housed in portable facilities (field truck and trailer) located near the western edge of the playa basin (Fig. 3A). Field operators of the rover, drill, robotic arm, and instruments issued control commands, supervised data collection, and then uploaded data daily to the mission server that was accessed via a portable satellite dish. Using commercially available file-sharing tools allowed for establishing easily configurable, low-latency repositories for raw data products uplinked by the field team and quick-look materials for communicating the strategic and tactical plans.

**FIG. 4.**
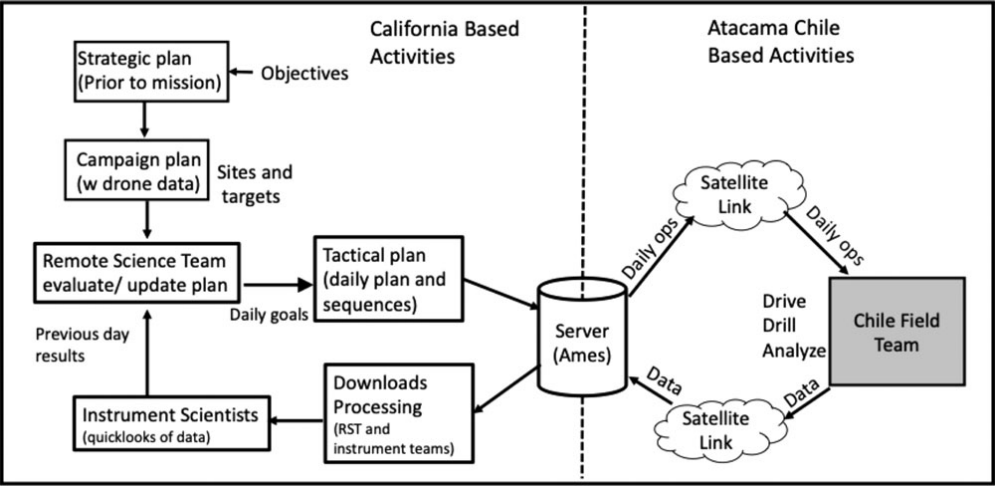
Flowchart illustrating the communication and data flow used in the test. The site was selected, and strategic plan was developed to meet the test objectives before deployment to Chile. Upon deployment, drone imaging was collected and used to refine the strategic plan to a campaign plan. During each day of the test the RST uploaded a tactical plan for the day's activities to a server that the field team accessed via satellite link. They performed the requested operations and uploaded the data to the same server where it was downloaded, processed, and interpreted by the RST and instrument scientists. At the start of each mission day, the RST reviewed the previous day's data then sent new instructions to the field in the day's tactical plan. RST = remote science team.

Figure 4 shows the flow of information used for strategic and tactical planning in daily operations modeled after the Mars 2020 rover mission operations adapted to an operational simulation in a field experiment. Each morning, the RST met to review and analyze the previous day's data acquired from the drilling and sample analysis. Then a planning meeting was held to produce the day's tactical plan and post it to the data server where it was accessed by the field team. The tactical plan was developed following the strategic plan template created before the mission start but modified based on the outcome of each previous day's operation and to accommodate specific requests from the instrument scientists. Supplementary Data document SOM1 shows the log of the executed tactical plans for each day of the test.

Tactical plans sent by the RST to the field team included a rover drive command with Universal Transverse Mercator coordinates for the desired destination. After each drive to a new site, the rover was instructed to acquire a 360° panorama by turning the rover in 60° increments (since navigation cameras were fixed on a mast), and then upload the panorama to the server. The RST was allowed 30 min to request another move or pick a drill spot visible in the panorama. Once the rover was moved to the selected location, the robotic arm was positioned to point its camera to image the location where the drill foot would contact the ground. These images were uploaded to the server, and the RST was allowed up to 30 min to examine the image and either request a move to adjust the location or give approval to start drilling.

Each drill sequence was commanded to acquire a sample from a specified depth that was reached by using the bite-sample approach. For each 10 cm of drilling, the drill returned to the surface, and subsurface material was removed from the auger flights by the cog wheel. By placing the scoop under the cog wheel, the material fell into the scoop, and when not from the desired sample depth, it was dumped in a pile away from the borehole. Once a sample from the desired depth was acquired in the scoop, an arm camera image was acquired and uploaded so that the RST could verify the scoop-contained sample before delivery to the instruments. The tactical plan also specified the instruments to receive samples, as not all instruments were required or able to analyze each sample.

Finally, after sample delivery, the arm camera was pointed at the drill string and an image was acquired to determine whether any material appeared to be stuck to it.

## 3. Results

### 3.1. Operational readiness test

Before the start of the mission, an operational readiness test (ORT) was performed at a previously visited site in Atacama, Chile, to verify all systems were working properly and refresh training for the RST and field team on mission protocols. This 1-day test immediately preceded the 6-day mission simulation. The ORT contributed to lessons learned and interpretation of mission results and so is discussed here. The test was held at an area known as “Green Parrot” that had been previously drilled with the ARADS equipment, so some members of the RST had prior experience drilling and analyzing samples there (Stoker *et al*., 2019). The site is covered with a 10–30-cm-thick halite deposit featuring meter-scale polygonal structures. Halite pinnacles form around the edges of the polygons that can extend up to 10–20 cm above the surface (see Fig. 3A in Glass *et al*., 2023).

Relatively diverse communities of microorganisms are found beneath the surface of the halite nodules that are thought to derive the moisture needed to survive from deliquescence and other minor wetting events (Wierzchos *et al*., 2006; Dávila *et al*., 2008). Underneath the halite at a depth of 40 cm, the subsurface is moist and sticks on the drill string even after removal by the cog wheel.

At the start of the ORT, the RST was provided with a high-resolution drone-acquired image of the Green Parrot site to select a drilling target. Then they created and uploaded a tactical plan to the server that included a GPS coordinate for the location to drill (−24.085500° latitude, −69.902833° longitude). The rover operators drove to the requested location and then acquired a 360° panorama with the navigation cameras. These data were uploaded to the server and the RST selected a direction to orient the rover. Images of the ground were then acquired with the arm camera, and the RST directed a small move command to reach an exact spot to deploy the drill. Next, an arm camera image of the location where the drill footprint would land was acquired. The RST reviewed the image and then requested drilling to 20 cm depth to acquire sample. Drilling was executed but no sample was acquired.

The field team reported that the drill foot had been placed at a location where a subsurface void was overlain by a thin halite shell. Surface and subsurface topographic variations such as these are common in the halite field at Green Parrot. Telemetry from the drill also showed very-low resistance during drilling, a clue that the drill encountered a subsurface void.

### 3.2. Imaging

**FIG. 5.**
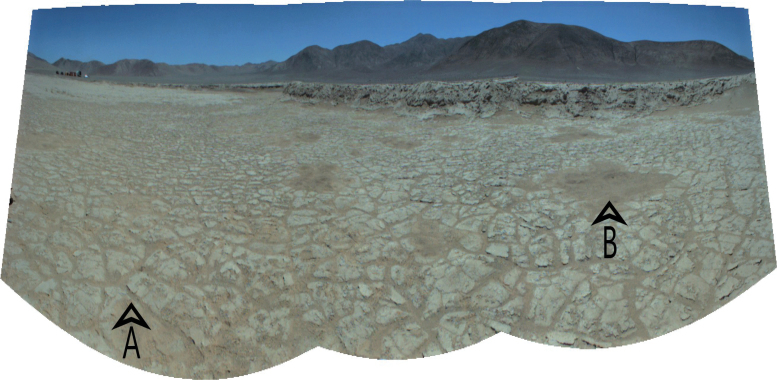
Orthorectified panorama created from mast camera images. The image center is facing North. “A” points to polygonal textures seen in this location. “B” points to depressions that are infilled with sediments.

On Sol 1 of the test, the rover arrived at Area 1 (−24.102089° latitude, −70.138063° longitude). The two mast cameras provide information for remote geological interpretation of the field site. The panorama of the area (Fig. 5) shows connected sedimentary ridges (uplifted borders) on the ground that form a self-organized network of polygons a few to tens of centimeters in diameter. Fine-grained windblown material had accumulated in the cracks between polygons, and larger meter sized depressions were infilled with dark fine-grained material. The drying of small salt ponds commonly produces a salt crust mixed with windblown sand or dust that settles in topographic lows or accumulates against the elevated rims of polygons. These are polygonal surface structures (PSS), which are known to exist on both Mars and Earth (Pina *et al*., 2008; DeCampo and Jones, 2014).

**FIG. 6.**
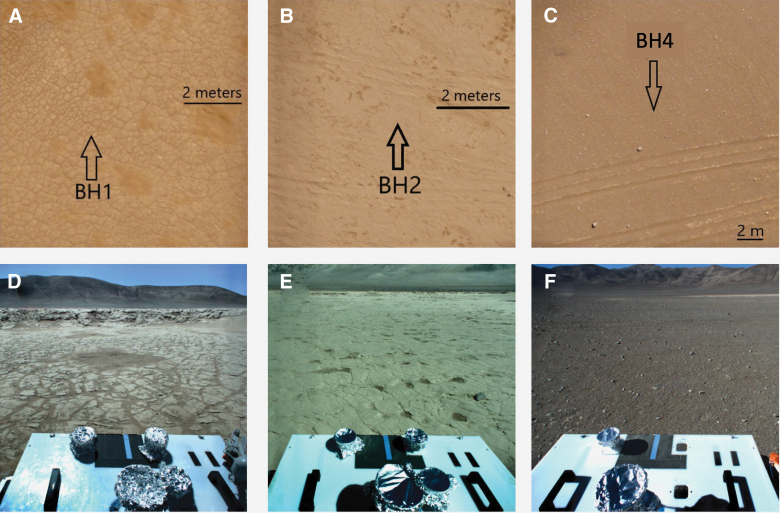
Aerial images **(A–C)** show each sample location on images taken by a drone. Polygonal patterns are only visible in **(A)**. Tracks seen in drone images were not made by the rover but by human-occupied vehicles. Images **(D–F)** were taken by the mast camera at the locations of holes in Areas 1, 2, and 3 respectively.

Figure 6 shows sections of the drone-acquired aerial images along with rover mast camera images of the three areas drilled showing their different surface textures. The RST interpreted the deeper basin (Area 1) as the youngest surface based on the well-preserved polygonal texture, with the shallower basin (Area 2) interpreted as older, based on the degree of erosion; polygonal structures are still visible but more eroded. Area 3 has a rocky desert pavement surface that takes a minimum of tens of thousands of years to develop (Bae Seong *et al*., 2016), so was interpreted as the oldest surface. The surface material at Area 1 and 2 was soft enough that rover tracks were visible in images acquired by the mast cameras, but the rover did not leave tracks in Area 3. The PSS are much less pronounced at Area 2 and absent at Area 3.

Taken together with topographic information (Fig. 3), the RST interpreted samples from Area 1 to represent material from a basin recently flooded with water, samples from Area 2 to represent a relatively higher basin that was flooded further in the past, and samples from Area 3 to represent the desert floor outside of the basins where no evidence of water modification was observed. The images taken by the arm camera that were meant to determine whether sample was acquired in the scoop before delivery showed an unexpected problem that prevented their intended use. On all occasions this activity was performed, the arm camera lens became covered with dust, and the images were obscured. Field observations showed that, when the scoop was placed next to the drill to capture cuttings as the drill is brought to the surface, fine material scraped off the drill was blown by the wind directly onto the lens of the arm camera where it accumulated and obscured the view.

**FIG. 7.**
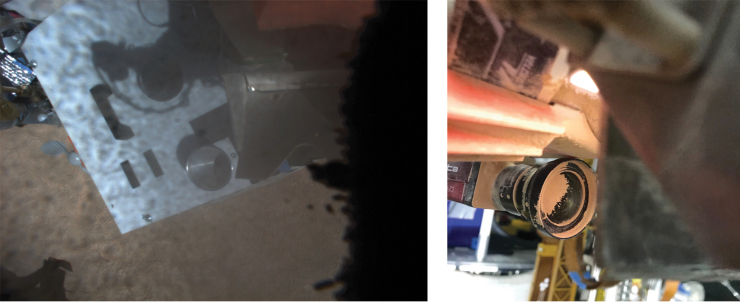
(Left) arm camera image acquired to determine whether sample is in the scoop. The image is obscured by dust on the lens. (Right) Photograph of arm camera lens collected after a drilling operation showing dust accumulated on the lens.

Figure 7 left panel shows an arm camera image that is largely obscured by dust, and Fig. 7 right panel shows a picture, taken by a field team member, of the camera lens nearly covered with dust. This incident illustrates the value of field testing to identify problems that might otherwise negatively impact a flight mission.

### 3.3. Sample analysis

Table 1 shows the areas, hole/sample names assigned by the RST, and locations of each sample analyzed. Table 1 also summarizes the success status from each sample analyzed by the instruments during the remote operation: “Yes” indicates a fully completed analysis, “Failed” indicates that the analysis was not successful, and “No” indicates the instrument did not receive or analyze this sample. Results from the previous day's sample analysis were presented to the RST by the instrument scientists during the daily planning meeting each morning of the test. These reports informed the tactical planning for the day's activities. While the RST requested samples to be analyzed from one specific depth, the field team collected all material coming to the surface from each 10 cm bite of drilling to be used for ground truth analysis and drilled each hole to a depth desired for that purpose.

**Table 1.** Samples Acquired and Analyzed Onboard the Rover

Date	Sample area	Hole/sample name	Location (Lat., Long.)	Sample depth, cm	WCL	SOLID	PISCES
September 15	1	1	−24.102089°, −70.138063°	10–20	Yes	Yes, biomolecules detected	Yes, AA detected
September 16	1	1A	−24.102089°, −70.138063°	40–50	No, not onboard	Yes, biomolecules detected	No (1)
September 17	2	2	−24.101642°, −70.138175°	10–20	Yes	Failed, not enough sample	Yes, AA detected
September 18	2	3	−24.101642°, −70.138175°	70–80	No, sample delivery failed	Yes, but no signal returned (2)	Yes, AA detected
September 19	3	4	−24.101238°, −70.140237°	10–20	No, not onboard	Yes, Nothing detected	Yes, nothing detected
September 20	1	5	−24.102089°, −70.138063°	70–73	Yes	No, not analyzed	No, not onboard

(1) PISCES team preferred to repeat the analysis of the September 15 sample to confirm results rather than another sample, but repeat analysis was unsuccessful (operator error).

(2) SOLID field engineer believed sample was not getting into the system and disassembled the instrument to clean out a potential clog after this run.

AA = amino acids; PISCES = Planetary *In Situ* Capillary Electrophoresis System; SOLID = Signs of Life Detector; WCL = Wet Chemistry Laboratory.

As mentioned previously, each RST-requested sample required a separate hole to be drilled because the equipment was stowed in a secure facility each night. To avoid contamination from human activities that occurred when the rover was prepared for driving back to base camp, the separate holes drilled in each area were usually separated by at least a meter. Details of sample analysis by the SOLID instrument along with extensive ground truth analysis of the samples is reported by Moreno-Paz *et al.* (2023). Mora *et al.* (2020) reported the results of the PISCES sample analysis.

### 3.4. Commands and timing

Table 2 shows the command cycles required for drill and arm operations for each hole drilled. For the purposes of this analysis, a command cycle occurs when a command is sent by a human operator to the control software running onboard the KREX2 rover, and an acknowledgment that the command has been received is sent back to the operator. Autonomous fault recovery routines invoked by the drill control software in response to off-nominal drilling conditions were not counted as command cycles since it was the onboard control software itself that invoked the command. This analysis does not include command cycles for rover operations because those are now routinely performed on flight missions. These data can be thought of as a command cycle analysis of remote semiautonomous drilling and sample handling operations onboard a stationary platform.

**Table 2.** Command Cycles Required to Accomplish the Drilling and Sample Delivery Operations

Hole	Date	Type	Command cycles											
			Start up	0–10 cm	10–20 cm		20–30 cm	30–40 cm	40–50 cm	50–60 cm	60–70 cm	70–80 cm	Shut down	Total
Hole #0A	September 13, 2019	In-Sim	3	5		3	2						2	15
		Out-of-Sim		2										2
Hole #0B	September 13, 2019	In-Sim	2	3		3	16						4	28
		Out-of-Sim		1		18								19
Hole #1	September 15, 2019	In-Sim	3	5		17							3	28
		Out-of-Sim												
Hole #1A	September 16, 2019	In-Sim	3	5		8	10	5	8	10			3	52
		Out-of-Sim												
Hole #2	September 17, 2019	In-Sim	1	5		13	11	5	5	5			4	49
		Out-of-Sim												
Hole #3	September 18, 2019	In-Sim	1	5		5	5	5	5	5	5	20	2	58
		Out-of-Sim												
Hole #4	September 19, 2019	In-Sim	3	5		11	5	5	5	5			4	43
		Out-of-Sim												
Hole #5	September 20, 2019	In-Sim	5	2		2	5	3	2	5	2	15	2	43
		Out-of-Sim										67		67

The analysis differentiates in-simulation from out-of-simulation command cycles. The latter are defined as command cycles for which the operator utilized information besides the data feeds available from the rover executive computer, such as an in-person visual inspection of the drill/arm or extra information relayed by field observers over handheld radio. Out-of-simulation command cycles always occurred in response to system faults that were detected by either the remote operator or field observer and were reacted to with immediate interventions to ensure the safe operation of equipment and people in the field.

Out-of-simulation command cycles for the drill and robotic arm only occurred for the two field ORT holes (#0A and #0B) undertaken before the start of the mission for test and training purposes, and for Hole #5 during the final bite when the drill became jammed at 72 cm. The jam occurred when a layer was encountered in which the drill could not move either forward or be pulled out. However, it was freed by cycles of forward and reverse drilling guided by feedback over radio by the field observer near the drill. More details of the jam and the procedure followed to release it are given below (Section 3.5).

Each command cycle shown in Table 2 is categorized as being either part of a particular depth-labeled bite (*e.g.,* 30–40 cm), or the payload startup/shutdown sequences. The beginning of a bite occurs when the robotic arm maneuvers the scoop end-effector into a position under the drill material chute to receive sample, and the end of the bite occurs when the scoop is fully emptied of sample. The exception to this rule is Hole #5, for which sample was collected without the use of the robotic arm for the first seven bites, and so, the number of command cycles per bite was on average much less than for the other holes. This bite-to-bite breakdown of command cycles allows for correlating the RST sample handling requirements of a particular bite to the command cycles that result from those requirements.

For instance, on Hole #0A, during the 0–10 cm bite, all the samples were collected by simply dumping it into a jar held by a field operator nearby where the rover was drilling, which means collection and delivery of the entire 0–10 cm bite only took five command cycles. Contrast that to Hole #1 during the 10–20 cm bite where the RST requested robotic arm delivery to all 3 instruments and a dump jar, which altogether took 17 command cycles. In many cases where the RST requested sample from a particular depth, drilling was continued after sample delivery and samples were collected for ground truth analysis by arm and scoop delivery to a clean glass jar. For example, in Hole #2, sample was collected and delivered to instruments from the 10 to 20 cm bite, requiring 18 command cycles, and then drilling was continued to 60 cm requiring 5 command cycles per bite.

**FIG. 8.**
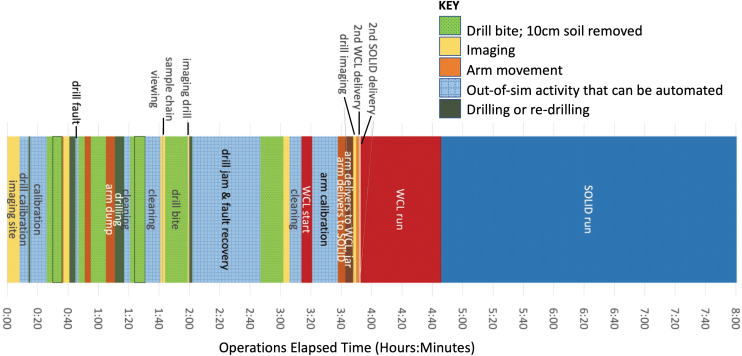
The operational time line for drilling the deepest hole (Hole 5) in Area 1.

A detailed event time and duration of all activities were recorded for each hole drilled. Figure 8 shows the operational timeline for the hole drilled on September 20 (Hole #5). The example shown is the worst case for the time required to complete the drilling and sampling operations that occurred during this test. The commanded depth requested by the RST (80 cm) was not reached because a layer was encountered at 72 cm in which the drill became stuck. Even with the extra time needed to free the jam, only 3.5 h of drill operations were required to accomplish drilling and sample collection in this hole. Drilling and sample delivery together typically required less than 30 min of operation time for each sample delivered to instruments (Glass *et al*., 2021).

### 3.5. Drilling

**FIG. 9.**
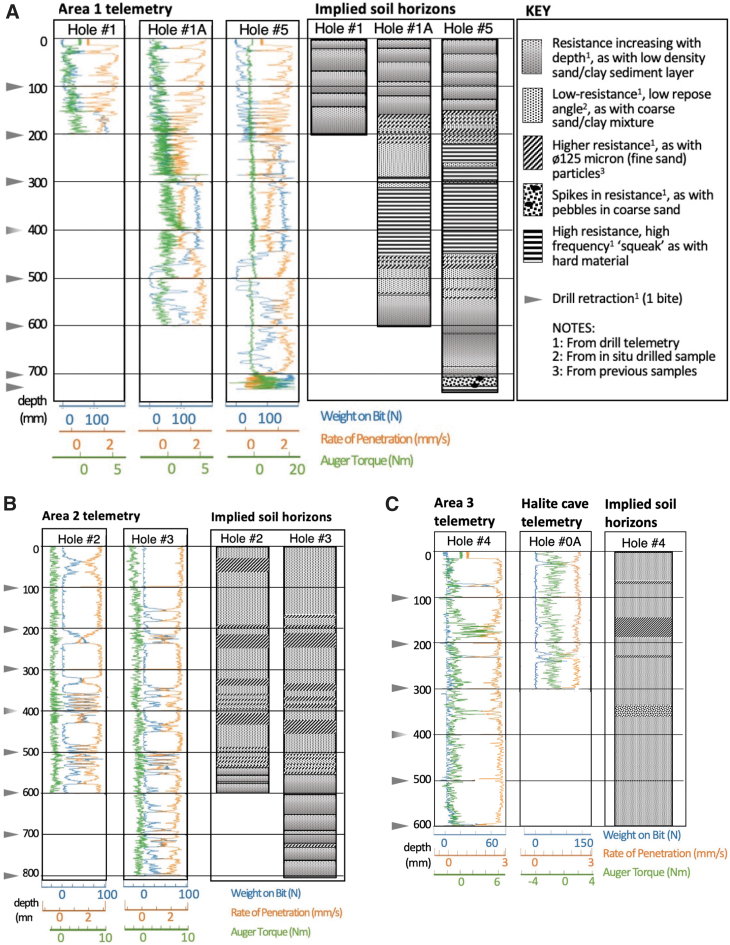
**(A)** Drill telemetry for the three holes drilled in Area 1 (left three plots), left to right is 20 cm hole, 60 cm hole, and 73 cm hole. The graphic on the right side shows the corresponding interpretation of the properties of the drilled materials. **(B)** The left two plots are drill telemetry for Area 2 Holes #2 and #3. The right two plots show interpreted material properties for these holes. **(C)** The left plot is drill telemetry for Area 3 Hole #4. The middle plot is drill telemetry from the ORT hole where the drill penetrated a thin halite shell at the surface that was hollow underneath, so encountered no material resistance. The materials properties inferred for Hole #4 are shown on right side. ORT = operational readiness test.

Drill telemetry parameters of WOB, rate of penetration (ROP), and auger torque were recorded along with depths where percussion occurred. These data were used to infer the subsurface properties and overall drilling conditions. Figure 9 shows the drill telemetry from all holes. The interpretations were guided by examination of telemetry from previous ARADS drillings in Atacama. Area 1 (Fig. 9A) from the surface to 20 cm depth shows a low resistance material with a fine scale (2–5 cm thick) repeated layering pattern indicating differences in induration. From 20 to 45 cm, the telemetry compares well with a denser gypsum-rich material encountered elsewhere in Atacama. Then from 45 to 52 cm a low resistance pattern appears again. From 52 to 70 cm, two layers are seen that repeat the near surface pattern. Finally, from 70 to 72 cm spikes in resistance are seen and squeaking noises were heard, as the drill hit the layer where it could make neither forward nor reverse progress.

Figure 9B shows drill telemetry from Area 2. The material properties down to 60 cm in Area 2 were quite different from those of Area 1. The ROP, WOB, and auger torque show that the material was more uniform to this depth; it was a coarse sand/clay mixture that was easy to drill through, interspersed with lenses of packed, fine sand that offered increased resistance. The ROP was typically 2–3 mm/s and the average auger torque near zero. From 60 to 80 cm, drill telemetry again showed low-to-high resistance layers consistent with sedimentary layering as seen in the 0–20 cm interval of Area 1. As Area 2 overlies Area 1, the sedimentary layers sensed near the bottom of Area 2 may extend downslope across the top of Area 1. Figure 9C shows the drill properties of Area 3 with very low resistance to drilling, in places comparable with Hole #0A in which nothing was drilled.

Most drill operations went smoothly except for the final bite of Hole #5 (70–80 cm) when the drill bit encountered a layer (suspected to be either cemented halite or a gravel layer) and became jammed at a depth of 72 cm. This jam, and the procedures used to free it, may provide useful insight for future planned drilling missions, and so, we include the following detailed description. Moments before the jam occurred, the control software detected a drastic slowdown in the auger spin and automatically invoked the jam recovery routine, which attempts to relieve pressure off the bottom of the borehole while also reaming the borehole walls by increasing the auger speed and retracting upward in brief increments. However, this automated recovery routine failed to prevent the jam, and the auger ceased spinning in the hole at 72 cm depth. Shortly after this, the drill motor's emergency stop button was hit by the on-sight observer to prevent motor burnout. Then actions were taken by both the remote operator and local observer to free the drill string.

First a retraction of the drill string by 2 cm while spinning the auger in reverse was attempted two different times, but only the second attempt succeeded in moving the drill string. With the drill string off the bottom of the hole, and freely rotating, another 2 cm retraction was attempted while the auger continued to spin. The local observer noticed that the z-stage did not appear to be moving upward; but the motor telemetry showed that the z-stage motor was still spinning, implying that the z-stage tether was being overtensioned, so the retraction was stopped at around 58 cm. To both relieve tension on the cable and determine if the blockage had been cleared, drilling to 80 cm was resumed. The drill again became jammed at 72 cm and was again emergency-stopped by the local observer.

Several attempts were then made to spin the auger both forward and backward in-place before the auger was again spinning freely. This minor success was accompanied by a loud squeaking noise, which is a typical symptom of a bind fault where a pebble becomes partially dislodged from the borehole wall and presses against the drill string causing unusually high friction. With hope that the downward motion would dislodge the binding material, drilling to 80 cm was again resumed. The drill was able to progress just a little bit deeper to 73 cm before the drill string became jammed again, requiring the drill motors to be stopped by the local observer again. Several attempts were then made to spin the auger in both the forward and reverse directions, which resulted in the auger momentarily spinning freely before it became jammed again several seconds later. At this point, another upward retraction of the drill string was attempted, and it advanced about 1 cm before the z-stage tether appeared overtensioned again, and so, the drill was locally stopped again. The field team then decided to focus on bringing the drill string out of the hole rather than continuing to drill to 80 cm. The drill's own native control software was used instead of the high-level rover control software to allow for lower level motor control. After about 20 more minutes of driving both the z-stage and auger in the back-and-forth directions, the drill finally began retracting nominally and returned to the surface. Finally, sample from the interval from 70 to 73 cm was captured and delivered to instruments.

## 4. Discussion

### 4.1. Operational performance

Despite the 4 hour time differences between RST and field team locations, the operation was able to meet the daily timelines and keep on schedule, thanks to the detailed planning and analysis of mission requirements performed to prepare for the field work, and disciplined adherence to preplanned schedules. The mission successfully made use of widely used commercial database management and team communication tools to manage and archive the data and to facilitate communication between field and science operations. This worked well to minimize miscommunications and disagreements between the two teams. The drill performed well in all holes without problems except for Area 1 Hole #5 (72+ cm) where the drill became stuck.

The command cycle analysis (Table 2), along with the episode of a stuck drill, provides important insight to help with operational planning for future drilling missions on other planets such as the upcoming VIPER and ExoMars missions. The operational protocols implemented here are similar to those that will be executed on the VIPER lunar mission where command cycles will occur rapidly. The VIPER operational timeline for drilling may resemble that shown in Fig. 8, excluding the sample analysis activities since VIPER does not collect or move samples into instruments.

However, on a Mars mission, a command cycle may take a full sol or more. Each 10 cm bite of drilling and capturing the cuttings in a scoop to move them away from the hole took five command cycles (Table 2). Moving cuttings away from the hole is needed to prevent them from falling back into the hole and mixing with the next bite, which could cause confusion and potentially cross-contamination between samples. Using the protocols and equipment implemented in this test, sample delivery to three instruments required 15–19 command cycles. The deepest delivered sample was from Hole 3 that required 55 command cycles to acquire and deliver sample from 80-cm depth to three instruments (Table 2). Clearly, if each command cycle requires a sol to complete, a Mars drilling mission would be a very slow process.

In this test, most of the command cycles were devoted to arm and scoop operation as opposed to drilling operations. Furthermore, multiple commands were not nested into automated sequences because the drill/arm operator felt that if anything went wrong during such a sequence, the time required to recover from the problem would far exceed manually commanding each step separately. However, the same issues would apply to a flight mission: once an error occurs, a lot of diagnosis and engineering time would be required to safely recover and resume normal operations. For Mars, there is a critical need for a high degree of automation of the sample handling to reduce the number of command cycles required, but with enough situational awareness that the operation can proceed safely.

The need for rapid sampling is compounded for volatile rich samples from the subsurface that may become quickly altered when exposed at the surface or when held in a scoop may stick to it or even disappear due to sublimation. Leaving sample in the scoop for even a single sol will not be acceptable for icy samples that are a priority target for searching for life on Mars (McKay *et al.*, 2013; McKay, 2020), and so, a single command sample delivery may be required. It is also important to verify that a sample has been delivered to an instrument before running the analysis without a sample present. On the Phoenix mission, icy samples became stuck in the scoop causing delivery failures. Samples also stuck on instrument inlets, sometimes resulting in failed analysis (Arvidson *et al*., 2009).

Our experiment, similar to the Phoenix mission, included a camera that could image into the scoop to verify the sample was present before delivery, but that camera lens became coated with fines that were blown onto it by the wind.

Automation and fault recovery for drilling will also be critical for a flight mission because only rapid fault detection can stop and protect a drill when a bind occurs. For example, lightspeed delays for Mars missions (tens of minutes) are much longer than the time required (seconds) for a drill to get stuck, so deep space drilling operations must be fully automated and fail-safe. The ARADS automated drilling control and executive software was designed to meet this need. It was built on lessons learned from over 15 years of drill autonomy software development. Software development began with the DAME drill (Glass *et al.*, 2008) using a simplified version of software later developed for the CRUX and Icebreaker drills (Glass *et al*., 2014). The ARADS software is a rewrite of CRUX's control software that incorporates lessons learned from testing the drill diagnostic procedures developed for both DAME and CRUX. It was rewritten in the NASA ARC-developed PLEXIL (plan execution language) to allow for a higher degree of modularity and on-the-fly modification or updates of diagnostics and recovery procedures. More detail on PLEXIL and sample acquisition and drilling robotics is given in Stucky *et al.* (2018) and Glass *et al.* (2021). The automated drill diagnostics and fault recovery were implemented in the field test but, for most of the test, it was not triggered because the drilling went smoothly in most holes that had only a few hard material encounters. It detected the jam in Hole #5 and the fault recovery mode was called but it was not successful at freeing the jam.

The lesson is that some situations may occur where operator commands may be necessary but detecting the problems and taking some action must be automated or the drill could fail catastrophically before the remote operators are aware of the problem.

### 4.2. Interpretation of the site from the sample analysis

If the hypotheses described in Section 2.4 are correct, SOLID and PISCES Instruments might be expected to show the strongest biological signatures in Area 1, weaker signatures in Area 2, and none or weakest in Area 3. However, due to problems experienced in the field, and the short duration of the test, not all samples were analyzed by both instruments (Table 1), complicating the comparison. Also, because the instruments used very different methodologies, a simple comparison between them is not practical. PISCES analyzed samples from all three areas but analyzed only the 10–20 cm sample from Area 1.

SOLID analyzed all the samples collected in all three areas, but the field engineer felt that lack of signal from Area 2 was due to a clogging problem with the instrument. The engineer cleaned out and refurbished the instrument before collection of the last two samples (Area 3 10–20 cm, and Area 1 70–73 cm). SOLID detected a significant signal from biomolecules in Area 1 in the 10–20 cm and 40–50 cm levels but did not analyze the 73 cm sample. The ground truth sample analysis with SOLID reported in Moreno-Paz et al. (2023) showed a decrease of biomarker signals from the 20 to 50 cm depth, consistent with the hypothesis put forward by the RST.

PISCES detected amino acids in all samples analyzed except at Area 3 where none was seen (Table 1). In Area 1, only the 20 cm sample was analyzed, but Area 2 samples were analyzed from 20 and 80 cm depth. The concentration of amino acids varied between samples, but there was no evidence that the overall amino acid concentration changed with depth. Only one amino acid (alanine) showed higher abundance in Area 1 than Area 2.

Taken together, results from SOLID and PISCES instruments indicate that biosignatures were present in Areas 1 and 2 but not in Area 3. SOLID and PISCES had corroborating results where they both successfully analyzed the same sample. Both results support the conclusion that biomolecules are correlated with aqueous activity in the basins and so most likely due to growth that occurs there when the area becomes wet as postulated by the RST.

### 4.3. RST interpretations compared with ground truth

In general, the RST correctly interpreted the nature of the study site using satellite and drone images and those taken by the rover as an area that becomes flooded after unusually large rainfall events. The RST also identified different areas of interest based on their potential to hold biomolecular signatures of life. Upon investigation of those areas by the rover and its science payload, it was confirmed that sediments in Areas 1 and 2, which were topographically lower and showed evidence of ponding after rain events, contained clear molecular signatures of life, including complex biomolecules and amino acids. In contrast, no detectable signatures were found in Area 3, a topographically higher area outside the basin covered with rocky desert pavement. These results confirm that small playas in topographic lows are sites of biological activity in the hyperarid core of the Atacama Desert after large but very unusual rainfall events. However, biology in soils surrounding those playas is much more subdued.

### 4.4. Subsurface structure from drill performance

In this article, we used the data acquired to monitor drill performance (WOB, auger torque, and ROP, Fig. 9) to infer the subsurface properties of the material drilled. This illustrates that the drill itself can be a powerful way to measure subsurface properties, but much remains to be done to enable interpretation of drill data on other planetary bodies. The interpretation we presented in Fig. 9 was based on patterns recognized from drilling many sites in Atacama with the ARADS drill. In a study focused on developing methods for interpreting drill data to identify icy deposits on the moon, a suite of cement and icy samples with different compressive strengths were drilled and the data used to train a machine learning algorithm to recognize the materials drilled (Joshi *et al*., 2020; Joshi, 2021). Machine learning automates pattern recognition but is dependent on sufficient training data to achieve accurate results.

To apply this approach to recognizing materials on other planets, it will be essential to obtain training data with engineering models of the flight drills and to drill into appropriate well-characterized formations. Along these lines, Peters *et al.* (2018) drilled terrestrial rock samples with an engineering model of the sample acquisition drill used on the Curiosity Mars rover, computed the energy required to drill these samples, and derived a functional relationship between the energy required for drilling and the compressive strength of the materials that was used to infer the strength of materials that were drilled on Mars.

## 5. Summary and Conclusions

The ARADS 2019 experiment in the Atacama Desert, Chile, was an end-to-end demonstration of a remotely directed robotic drilling mission to search for biomolecular evidence of life that was successfully conducted in a biologically lean Mars analog site. The sample analysis instruments, as well as the drill and sample delivery systems used in the test, were relatively mature flight prototypes. The preparation and planning for the mission and the operations performed mimicked those used on flight missions. The location selected for the experiment and the drilling targets within it were selected by a science team based on remotely acquired data and addressed hypotheses they developed based strictly on that data.

During the mission, the science team in California created and uploaded to a server a daily tactical plan for drilling and sample analysis. The drill, sample delivery arm, and sample analysis instruments were remotely operated by engineers in Chile that sent commands wirelessly to the robotic systems to accomplish the instructions they received. Six consecutive daily operations of a drilling mission were simulated in this manner. The mission successfully drilled in three areas separated by hundreds of meters. The areas drilled included (1) a deep basin showing evidence of aqueous activity within the relatively recent past, (2) a shallower basin showing evidence of aqueous activity that occurred further in the past than the deeper basin, and (3) the area surrounding the basins that showed no evidence of modification by water. The two basins were each drilled to ∼80 cm depth with samples collected at shallow (10–20 cm), mid (50–60 cm), and deep (70–80 cm) depths. The area outside the basin was drilled and sample collected from a shallow (10–20 cm) depth. Samples from all areas were analyzed by two different flight prototype instruments with complementary capabilities for detecting biologically produced molecules: SOLID that uses immunoassay to identify biomolecular evidence of life and PISCES that uses microchip electrophoresis to detect amino acids.

Sample analysis from the SOLID instrument detected the presence of various biomolecules in the deep basin samples, with the strongest signatures coming from the 10- to 20-cm-depth sample. No signatures were detected in samples from the shallower basin although technical problems occurred that may have prevented successful analysis of those samples. SOLID did not detect evidence of biosignatures in the sample collected outside the basin. Sample analysis from the PISCES instrument showed that amino acids were detected at all depths analyzed in both deep and shallow basins, but none was detected from the sample collected from the area outside the basins.

The number of command cycles to accomplish the mission objectives was tracked. The deepest sample that was acquired with fully remote operations required 55 command cycles to drill to 80 cm depth with sample collection and delivery to 3 instruments. Drilling was performed using the bite-sampling approach where the drill is brought to the surface to clear the cuttings from the hole after each 10 cm of drilling.

Drill telemetry was used to interpret the subsurface stratigraphy of materials that were drilled, showing fine-scale layers of sand/clay sediments interspersed with some layers of harder material in the basins and a uniform subsurface composed of coarse-to-fine sand in the region outside the basins.

In terms of protocols and technology for life detection on other planetary bodies, the ARADS campaign yielded the following important takeaway lessons:

(1) Contextual information is critical for sample site selection and data interpretation. This is particularly relevant in environments where evidence of life is expected to be heterogeneously distributed both on the surface and in the subsurface. In this scenario, surface mobility and vertical reach greatly increase the likelihood of a positive detection.
(2) Technologies for end-to-end sample acquisition, handling, and sample analysis currently exist that can find biomolecular evidence of life in one of the most extreme and biologically sparse environments on Earth. The technologies are at a sufficient level of maturity to be considered for future flight missions.
(3) The combination of multiple independent analytical tools that target different types of biosignatures (*e.g.*, complex biomolecules and amino acids) is a powerful approach to search for evidence of life. Independent lines of evidence from different instruments strengthen the interpretation of signals (particularly biological ones). Results from different instruments can also complement each other in the event of a null result from one instrument, or of instrument failure.
(4) Robotic drilling and sample handling are key technologies that are substantially less mature than roving but are crucial in life search scenarios for Mars as was emphasized in the 2020 Decadal Survey (National Academies of Sciences, Engineering, and Medicine, 2022). Tests in high-fidelity Mars analog environments help illuminate the operational complexities of deep drilling and should enable better performance on future missions.

## Supplementary Material

Supplemental data

## Abbreviations Used

AA: amino acids
ARADS: Atacama Rover Astrobiology Drilling Studies
ARC: Ames Research Center
CAB: Centro de Astrobiología
CL: chemical laptop
CP: chronopotentiometry
LITA: life in the Atacama
MECA: Microscopy Electrochemistry Conductivity Analyzer
ORT: operational readiness test
PISCES: Planetary *In Situ* Capillary Electrophoresis System
PSS: polygonal surface structures
PSTAR: Planetary Science and Technology through Analog Research
ROP: rate of penetration
RST: remote science team
SOLID: Signs of Life Detector
TRIDENT: The Regolith and Ice Drill for Exploration of New Terrains
UV: ultraviolet
VERVE: Visual Environment for Remote Virtual Exploration
VIPER: Volatiles Investigating Polar Exploration Rover
WCL: Wet Chemistry Laboratory
WOB: weight on bit

## Notes

Associate Editor: Christopher McKay
