# Supplementary material for: A Mission Simulating the Search for Life on Mars with Automated Drilling, Sample Handling, and Life Detection Instruments Performed in the Hyperarid Core of the Atacama Desert, Chile
Source: Astrobiology. 2023 Dec 20;23(12):1284–302. doi: 10.1089/ast.2022.0055 (PMC10750310; doi:10.1089/ast.2022.0055)
Supplement: Supplemental data [file Suppl_Data.pdf]

Supporting Online Material
Record of Tactical Commands

START SOL 1 TACTICAL PLAN
Process : Move to Location
Begin Command
"*Remote Team TO DO :
Upload Annotated Drone Image with
desired GPS location, preferred path and any Keep Off Zones if required*"
GPS Coordinate (UTM) : UTM 19J 384310.65 m E, 7333977.91 m S
Preferred orientation (Facing) : North
Comment :
End Command

SITE IMAGE GOES HERE

Begin Command
*Image Request(s) : * Nav-Cam Panorama (PANORAMA - 3 IMAGES)
Rover Data Product Number: RDPXXX RDP015
Preferred Orientation : North
Comment :
End Command

Begin Command
*Image Request(s) : * Look at Drill Site
Rover Data Product Number: RDPXXX RDP016
Preferred Orientation : N/A
Comment :
End Command

Begin Command
*Image Request(s) : * Wrist camera Near Field View (1 Image)
Rover Data Product Number: RDPXXX RDP017
Preferred Orientation : N/A
Comment :
End Command

Process : Perform Drilling Sequence
Begin Command
*Collect Sample at Depth range : * 10-20cm
Image Request : Sample Acquisition confirmation
End Command

Process : Sample Delivery

Begin Command
Skimming : YES
WCL : Yes
PISCES : Yes
SOLID : Yes
Dump Remainder in Jar (for LiTMS) : YES
End Command

Begin Command
*Image Request(s) : * Sample Delivery Confirmation
Rover Data Product Number: RDPXXX RDP018
Preferred Orientation : N/A
Comment : Remove funnel on jar prior image.
End Command

Begin Command
*Image Request(s) : * Nav-Cam Image Aquisiton (1 Image)
Rover Data Product Number: RDPXXX RDP019
Preferred Orientation : N/A
Comment : Remove funnel on jar prior image.
End Command
Begin Command
*Image Request(s) : * Look at Drill String
Rover Data Product Number: RDPXXX RDP020
Preferred Orientation : N/A
Comment :
End Command

Process : Sample Analysis
Begin Sample Anaylsis as Follows
WCL start at t=0
SOLID start at completion of WCL
LiTMS N/A
PISCES start after completion of SOLID (timing at discretion of field team)
Comments

END SOL 1 TACTICAL PLAN

START SOL 2 TACTICAL PLAN

Process : Perform Drilling Sequence
Begin Command
*Collect Sample at Depth range : * 10-20cm
Image Request : Sample Acquisition confirmation
Comment: Keep arm camera away from drill until time to acquire sample
End Command

Process : Sample Delivery
Begin Command
Skimming : YES
WCL : NO
PISCES : Yes
SOLID : NO
Dump Remainder in Jar (for LiTMS) : YES
End Command

Begin Command
*Image Request(s) : * Sample Delivery Confirmation
Rover Data Product Number: RDPXXX RDP021
Preferred Orientation : N/A
Comment :

Begin Command
*Image Request(s) : * Nav-Cam Image Acquisition (1 Image)
Rover Data Product Number: RDPXXX RDP022
Preferred Orientation : N/A
Comment :
End Command

Begin Command
*Image Request(s) : * Look at Drill String
Rover Data Product Number: RDPXXX RDP023
Preferred Orientation : N/A
Comment :
End Command

Process : Perform Drilling Sequence
Begin Command

*Collect Sample at Depth range : * 40-50cm
Image Request : Sample Acquisition confirmation
Comment: Keep arm camera away from drill until time to acquire sample
End Command

Process : Sample Delivery
Begin Command
Skimming : YES
WCL : NO
PISCES : NO
SOLID : Yes
Dump Remainder in Jar (for LiTMS) : YES
End Command

Begin Command
*Image Request(s) : * Sample Delivery Confirmation
Rover Data Product Number: RDPXXX RDP024
Preferred Orientation : N/A
Comment :
End Command

Begin Command
*Image Request(s) : * Nav-Cam Image Acquisition (1 Image)
Rover Data Product Number: RDPXXX RDP025
Preferred Orientation : N/A
Comment :
End Command

Begin Command
*Image Request(s) : * Look at Drill String
Rover Data Product Number: RDPXXX RDP026
Preferred Orientation : N/A
Comment :
End Command

Process : Sample Analysis
Begin Sample Analysis as Follows
WCL Do not run
SOLID start at t+0
LiTMS N/A
PISCES start after completion of SOLID (timing at discretion of field team)
Comments SOLID should be processing the 50cm bite, and PISCES the 20cm bite.
End Command
END SOL 2 TACTICAL PLAN

START SOL 3 TACTICAL PLAN

Process : Move to Location
Begin Command
"*Remote Team TO DO :
Upload Annotated Drone Image with
desired GPS location, preferred path and any Keep Off Zones if required*"
GPS Coordinate (UTM) : UTM 19J 384321.00 m E, 7334051.00 m S
Preferred orientation (Facing) : North
Rover Data Product Number: RDPXXX RDP027
Comment : Collect Navcam images approximately every 10m
End Command

SITE IMAGE GOES HERE

Begin Command
*Image Request(s) : * Nav-Cam Panorama (PANORAMA - 3 IMAGES)
Rover Data Product Number: RDPXXX RDP028
Preferred Orientation : North
Comment :
End Command

Begin Command
*Image Request(s) : * Nav-Cam Panorama (PANORAMA - 3 IMAGES)
Rover Data Product Number: RDPXXX RDP029
Preferred Orientation : South
Comment :
End Command

Process : Move to Drill Target
Begin Command
"*Remote Team TO DO :
Annotate then upload images received from rover with a red square to identify preferred drilling spot*"
End Command

DRILL TARGET IMAGE GOES HERE

Begin Command
*Image Request(s) : * Look at Drill Site
Rover Data Product Number: RDPXXX RDP030
Preferred Orientation : N/A
Comment :
End Command

Begin Command
*Image Request(s) : * Wrist camera Near Field View (1 Image)
Rover Data Product Number: RDPXXX RDP031
Preferred Orientation : N/A
Comment :
End Command

Process : Perform Drilling Sequence
Begin Command
*Collect Sample at Depth range : * 10-20cm
Image Request : Sample Acquisition confirmation
Rover Data Product Number: RDPXXX RDP 032A
Comment Keep arm camera away from drill until sample collection.
End Command

Process : Sample Delivery
Begin Command
Skimming : YES
WCL : Yes
PISCES : Yes
SOLID : Yes
Dump Remainder in Jar (for LiTMS) : YES
End Command

Begin Command
*Image Request(s) : * Sample Delivery Confirmation
Rover Data Product Number: RDPXXX RDP032
Preferred Orientation : N/A
Comment : Remove funnel on jar prior image.
End Command

Begin Command
*Image Request(s) : * Nav-Cam Image Acquisition (1 Image)
Rover Data Product Number: RDPXXX RDP033
Preferred Orientation : N/A
Comment : Remove funnel on jar prior image.
End Command

Begin Command
*Image Request(s) : * Look at Drill String
Rover Data Product Number: RDPXXX RDP034
Preferred Orientation : N/A
Comment :

End Command

Process : Sample Analysis
Begin Sample Analysis as Follows
WCL start at t+0
SOLID start at completion of WCL
LiTMS N/A
PISCES start after completion of SOLID (timing at discretion of field team)
Comments
End Command

END SOL 3 TACTICAL PLAN

START SOL 4 TACTICAL PLAN

Process : Perform Drilling Sequence
Begin Command
*Collect Sample at Depth range : * 70-80cm
Image Request : Sample Acquisition confirmation
Rover Data Product Number: RDPXXX RDP035A
Comment At discretion of field team, remote science team will accept lesser sample depth.
End Command

Process : Sample Delivery
Begin Command
Skimming : YES
WCL : Yes
PISCES : Yes
SOLID : Yes
Dump Remainder in Jar (for LiTMS) : YES
End Command

Begin Command
*Image Request(s) : * Sample Delivery Confirmation
Rover Data Product Number: RDPXXX RDP035
Preferred Orientation : N/A
Comment :
End Command

Begin Command
*Image Request(s) : * Nav-Cam Image Acquisition (1 Image)
Rover Data Product Number: RDPXXX RDP036
Preferred Orientation : N/A
Comment :
End Command

Begin Command
*Image Request(s) : * Look at Drill String
Rover Data Product Number: RDPXXX RDP037
Preferred Orientation : N/A
Comment :
End Command

Process : Sample Analysis
Begin Sample Analysis as Follows
WCL start at t+0
SOLID start at completion of WCL

LiTMS N/A
PISCES start after completion of SOLID (timing at discretion of field team)
Comments
End Command

END SOL 4 TACTICAL PLAN

START SOL 5 TACTICAL PLAN

Process :   Move to Location
Begin Command
"*Remote Team TO DO :
Upload Annotated Drone Image with
desired GPS location, preferred path and any Keep Off Zones if required*"
GPS Coordinate (UTM) :   UTM 19J 384111.00 m E, 7334094.00 m S
Preferred orientation (Facing) :   North
Comment :   Collect Navcam images approximately every 10m if possible
End Command

SITE IMAGE GOES HERE

Begin Command
*Image Request(s) : *   Nav-Cam Panorama (PANORAMA - 3 IMAGES)
Rover Data Product Number: RDPXXX   RDP038
Preferred Orientation :   North
Comment :   Part 1 of 2 for 360 Panorama
End Command

Begin Command
*Image Request(s) : *   Nav-Cam Panorama (PANORAMA - 3 IMAGES)
Rover Data Product Number: RDPXXX   RDP039
Preferred Orientation :   South
Comment :   Part 2 of 2 for 360 Panorama
End Command

Process :   Adjustment of Drill Target
Begin Command
"*Remote Team TO DO :
Annotate then upload images received from rover with a red square to identify preferred drilling spot*"
End Command

Begin Command
*Image Request(s) : *   Look at Drill Site
Rover Data Product Number: RDPXXX   RDP040
Preferred Orientation :   N/A
End Command

Begin Command
*Image Request(s) : *   Wrist camera Near Field View (1 Image)
Rover Data Product Number: RDPXXX   RDP041

Preferred Orientation : N/A
Comment :
End Command

Process : Perform Drilling Sequence
Begin Command
*Collect Sample at Depth range : * 10-20cm
Image Request : Sample Acquisition confirmation
Rover Data Product Number: RDPXXX RDP042A
Comment We would like you to dill a 60cm profile while waiting for SOLID to be remounted. We want to see what material has collected on the arm camera lens. If possible please position the arm so the camera lens is visible in the nav cam FOV after each sample bite is collected. Once SOLID is re-mounted and if time permits, please deliver the sample bite specified above. Field team can override this request if it is deemed unsafe or impractical.
End Command

Process : Sample Delivery
Begin Command
Skimming : YES
WCL : No
PISCES : Yes
SOLID : Yes
Dump Remainder in Jar (for LiTMS) : YES
End Command

Begin Command
*Image Request(s) : * Sample Delivery Confirmation
Rover Data Product Number: RDPXXX RDP042
Preferred Orientation : N/A
Comment :
End Command

Begin Command
*Image Request(s) : * Nav-Cam Image Acquisition (1 Image)
Rover Data Product Number: RDPXXX RDP043
Preferred Orientation : N/A
Comment :
End Command

Begin Command
*Image Request(s) : * Look at Drill String
Rover Data Product Number: RDPXXX RDP044
Preferred Orientation : N/A
Comment :

End Command

Process : Sample Analysis
Begin Sample Analysis as Follows
WCL N/A
SOLID start t+0
LiTMS N/A
PISCES start after completion of SOLID (timing at discretion of field team)
Comments
End Command

END SOL 5 TACTICAL PLAN

START SOL 6 TACTICAL PLAN

Process :   Move to Location
Begin Command
"*Remote Team TO DO :
Upload Annotated Drone Image with
desired GPS location, preferred path and any Keep Off Zones if required*"
GPS Coordinate (UTM) :   UTM 19J 384310.65 m E, 7333977.91 m S
Preferred orientation (Facing) :   North
Comment :   Collect Navcam images approximately every 10m if possible
End Command

SITE IMAGE GOES HERE

Begin Command
*Image Request(s) : *   Nav-Cam Panorama (PANORAMA - 3 IMAGES)
Rover Data Product Number: RDPXXX   RDP045
Preferred Orientation :   North
Comment :   Part 1 of 2 for 360 Panorama
End Command

Begin Command
*Image Request(s) : *   Nav-Cam Panorama (PANORAMA - 3 IMAGES)
Rover Data Product Number: RDPXXX   RDP046
Preferred Orientation :   South
Comment :   Part 2 of 2 for 360 Panorama
End Command

Begin Command
*Image Request(s) : *   Look at Drill Site
Rover Data Product Number: RDPXXX   RDP047
Preferred Orientation :   N/A
End Command

Begin Command
*Image Request(s) : *   Wrist camera Near Field View (1 Image)
Rover Data Product Number: RDPXXX   RDP048
Preferred Orientation :   N/A
Comment :
End Command

Process :   Perform Drilling Sequence
Begin Command
*Collect Sample at Depth range : *   50-60cm

Image Request : Look at Drill String
Rover Data Product Number: RDPXXX RDP049
Comment RST does not want to collect this sample, just drill and look at drill string.
End Command

Process : Perform Drilling Sequence
Begin Command
*Collect Sample at Depth range : * 60-70cm
Image Request : Look at Drill String
Rover Data Product Number: RDPXXX RDP050
Comment RTS does not want to collect this sample, just drill and look at drill string
End Command

Process : Perform Drilling Sequence
Begin Command
*Collect Sample at Depth range : * 70-80cm
Image Request : Sample Acquisition Confirmation
Rover Data Product Number: RDPXXX RDP051
Comment RTS Wants this sample
End Command

Process : Sample Delivery
Begin Command
Skimming : YES
WCL : Yes
PISCES : No
SOLID : Yes
Dump Remainder in Jar (for LiTMS) : YES
End Command

Begin Command
*Image Request(s) : * Sample Delivery Confirmation
Rover Data Product Number: RDPXXX RDP052
Preferred Orientation : N/A
Comment :
End Command

Begin Command
*Image Request(s) : * Nav-Cam Image Acquisition (1 Image)
Rover Data Product Number: RDPXXX RDP053
Preferred Orientation : N/A
Comment :
End Command

Begin Command
*Image Request(s) : * Look at Drill String
Rover Data Product Number: RDPXXX RDP054
Preferred Orientation : N/A
Comment :
End Command

Process : Sample Analysis
Begin Sample Analysis as Follows
WCL start t+0
SOLID start after completion of WCL
LiTMS N/A
PISCES N/A
Comments
End Command

END SOL 6 TACTICAL PLAN
